# Amyloid-β drives a type-1 interferon mediated neuro-inflammatory response in Alzheimer’s disease

**DOI:** 10.1186/1750-1326-8-S1-P29

**Published:** 2013-09-13

**Authors:** Myles Minter, Juliet Taylor, Moses Zhang, Paul Adlard, Peter Crack

**Affiliations:** 1 Department of Pharmacology & Therapeutics, University of Melbourne, Parkville, Victoria, Australia; 2 Synaptic Neurobiology Laboratory, Mental Health Research Institute, Parkville, Victoria, Australia

## Background

Neuro-inflammation has been implicated in the progression of both acute and chronic neurological diseases. Resident cells of the central nervous system (CNS) detect soluble amyloid-β (Aβ) through the toll-like receptors (TLRs), involving Myd88 and interferon regulatory factor (IRF) signalling, and triggers pro-inflammatory cytokine release. Type-1 interferons (IFNs) are master regulators of the pro-inflammatory cytokine response, however, their CNS function remains largely unclear. Type-1 IFNs bind their cognate receptor IFNAR1, activating the JAK-STAT signalling pathway. Significantly, this cascade has been implicated as a mediator soluble Aβ1-42-induced toxicity [1]. We have previously demonstrated that removal of IFNAR1, contributes to neuro-protection following Aβ1-42 insult, decreasing type-1 IFN production and apoptosis. This study investigated a role for type-1 IFNs in an AD mouse model and utilised an in vitro approach to analyse TLR signalling as a potential production pathway.

## Materials and methods

Total protein brain extracts were prepared from wild-type and B6C3-TgAPP_swe_, PSEN1_ΔE9_ (APP/PS1) mice (9 months) with IFNα levels measured by ELISA and phosphorylated Stat-3 (p-Stat-3) by Western blot. Immunohistochemistry was performed on aged brains (13 months) using p-Stat-3, FOX-3a and GFAP antibodies. Primary wild-type and Myd88-/- murine neurons were treated with 2.5µM Aβ1-42 for 24-72 hours and Q-PCR analysed IFNα, IFNβ, IL-1β and IL-6 expression. BE(2)-M17 human neuroblastoma cells were transfected stably with an IRF7 or transiently with an IRF3 knockdown (KD) shRNA plasmid or negative control (NC) plasmid and subjected to 7.5µM Aβ1-42 for 24-96 hours. Q-PCR analysed IFNα and IFNβ expression, Western blot determined p-Stat-3 expression, and an MTS assay assessed cell viability.

## Results

APP/PS1 brains (9 months) demonstrated a significant 2-fold increase in IFNα protein levels compared to aged matched controls (n=4, P<0.05). Western blot analysis confirmed robust p-Stat-3 in these APP/PS1 brains (n=4). Immunohistochemistry of 13 month brains confirmed co-localisation of p-Stat-3 with the neuronal marker, FOX-3a, not the glial marker, GFAP (n=5). Following Aβ1-42 treatment Myd88-/- neurons showed reduced IFNα (5.3-fold), IFNβ (2.2-fold), IL-1β (2.1-fold) and IL-6 (27.2-fold) expression (n=2, 24hrs). IRF7 KD in M17 cells did not alter the Aβ1-42-induced type-1 IFN response with no change in Aβ1-42 toxicity compared to NC cells (n=3). However, M17 IRF3 KD cells demonstrated reduced IFNα (3.7-fold) and IFNβ (2.3-fold) expression following Aβ1-42 insult (n=3, P<0.05, 24hrs). This correlated with reduced Aβ-induced toxicity compared to NC cells (97.7% vs. 65.2%, 96hrs, n=5, P<0.05).

## Conclusions

This study supports a role for type-1 IFN signalling in the pro-inflammatory response generated by Aβ. The data suggests that Aβ-induced type-1 IFN production is TLR-regulated and occurs through an IRF3-dependent mechanism. Modulating these pathways may be beneficial in reducing type-1 IFN production and neuro-inflammation, consequently limiting neuronal damage in AD.
